# YOUNG PEOPLE FEEL WISE, OLD PEOPLE FEEL ENERGETIC: COMPARING AGE STEREOTYPES AND SELF-EVALUATIONS ACROSS ADULTHOOD

**DOI:** 10.1093/geroni/igz038.2898

**Published:** 2019-11-08

**Authors:** Anna E Kornadt, Catherine E Bowen, Svenja M Spuling, Maja Wiest

**Affiliations:** 1 Bielefeld University, Bielefeld, Germany; 2 Independent Researcher, Vienna, Wien, Austria; 3 German Centre of Gerontology (DZA), Berlin, Berlin, Germany; 4 Free University Berlin, Berlin, Berlin, Germany

## Abstract

Using questionnaire data from the MIDUS study (N=6.325) we examined the extent to which people in their late 20s, 40s, and 60s think that positive stereotypic “old” and “young” characteristics describe themselves, their age peers, and other age groups. A constellation of “old” characteristics (e.g., wise, caring, calm) was seen as more descriptive of older adults, while a constellation of “young” characteristics (e.g., healthy, energetic) was seen as more descriptive of younger adults. Self-evaluations were highly positive and largely consistent across age groups. Compared to their age peers, younger adults saw themselves as having as many positive “young” characteristics but more positive “old” characteristics whereas older adults saw themselves as having more positive “young” characteristics but fewer positive “old” characteristics. The results support the stability of the aging self despite the existence of age stereotypes and the role of negative age stereotypes as a frame of reference for making self-evaluations.

